# CDK Regulation of Meiosis: Lessons from *S. cerevisiae* and *S. pombe*

**DOI:** 10.3390/genes11070723

**Published:** 2020-06-29

**Authors:** Anne M. MacKenzie, Soni Lacefield

**Affiliations:** Department of Biology, Indiana University, 1001 E. Third Street, Bloomington, IN 47405, USA; mackenza@indiana.edu

**Keywords:** meiosis, Cyclin-Dependent Kinase, CDK, cyclin, APC/C, budding yeast, fission yeast, chromosome segregation

## Abstract

Meiotic progression requires precise orchestration, such that one round of DNA replication is followed by two meiotic divisions. The order and timing of meiotic events is controlled through the modulation of the phosphorylation state of proteins. Key components of this phospho-regulatory system include cyclin-dependent kinase (CDK) and its cyclin regulatory subunits. Over the past two decades, studies in budding and fission yeast have greatly informed our understanding of the role of CDK in meiotic regulation. In this review, we provide an overview of how CDK controls meiotic events in both budding and fission yeast. We discuss mechanisms of CDK regulation through post-translational modifications and changes in the levels of cyclins. Finally, we highlight the similarities and differences in CDK regulation between the two yeast species. Since CDK and many meiotic regulators are highly conserved, the findings in budding and fission yeasts have revealed conserved mechanisms of meiotic regulation among eukaryotes.

## 1. Introduction

Control of the eukaryotic cell cycle occurs through the modulation of phosphorylation states of proteins that trigger specific events. At the forefront of this phospho-regulation are the cyclin-dependent kinases (CDKs), whose oscillatory activity results in a large number of phosphorylations that change the activation state of their substrates [1,2]. Ultimately, CDK controls the cell cycle by regulating many processes including DNA replication in S phase (synthesis) and chromosome segregation in M phase (mitosis). Similarly, CDK is also essential for meiotic regulation, with additional roles in ensuring meiosis-specific events.

The genes encoding CDKs were originally discovered in landmark genetic screens performed by Lee Hartwell and Paul Nurse in budding and fission yeast, respectively [3,4,5]. In addition, further analysis of CDK regulation in these model organisms proves to be foundational for our understanding of the eukaryotic cell cycle. Both yeasts encode one CDK, Cdk1, that governs both the mitotic and meiotic cell cycles. Cdk1 is present throughout the cell cycle, but its oscillatory activity is dependent on the regulatory subunits known as cyclins [1,2]. Cyclins stimulate the activity of CDK and specify substrates for phosphorylation. In addition, Cdk1 and cyclins can be regulated by post-translational mechanisms and through the binding of inhibitors. 

There are many similarities to the regulation of CDK activity in the budding and fission yeast mitotic cell cycle. In G1 phase (Gap 1), CDK activity is low [6,7,8,9]. With signals such as cell growth, the G1 cyclin levels rise and bind CDK (G1-CDK). G1-CDK phosphorylates and inhibits the CDK inhibitors and the Anaphase Promoting Complex/Cyclosome (APC/C), a ubiquitin ligase that targets the S and M phase cyclins for proteasomal degradation [10,11,12,13,14,15]. With the inhibition of the APC/C, S phase cyclins increase and bind CDK (S-CDK). Phosphorylation of S-CDK substrates leads to the initiation of DNA replication and S phase progression [16,17]. M phase cyclins are expressed as cells exit S phase [18,19]. In fission yeast, CDK bound to M phase cyclins (M-CDK) is inhibited by the Wee1 and Mik1 kinases, which place an inhibitory phosphorylation on CDK [20,21,22]. The inhibition of CDK allows further growth in G2 (Gap 2) before transitioning into M phase. Once cells are ready to exit G2, Cdc25 phosphatase removes the inhibitory phosphorylation, unleashing active M-CDK [22,23,24,25]. Budding yeast has a Wee1 homolog called Swe1. However, Swe1 is not thought to have as important of a role in normal cell cycle progression [26,27]. Instead, M-CDK is activated with the production of the M phase cyclins. M-CDK activity controls many events in M phase such as chromosome condensation, spindle assembly, and chromosome attachment on the bipolar spindle [28,29,30]. M-CDK also activates its inhibitor, the APC/C, allowing the cells to transition into anaphase [31,32]. CDK activity declines in anaphase because the cyclins are degraded, CDK inhibitors are activated, and the phosphatase Cdc14 in budding yeast, or Clp1 in fission yeast, removes the phosphorylations from CDK substrates [28,33,34,35,36,37]. Cells will exit M phase and enter into G1 with low CDK activity.

**Figure 1:**
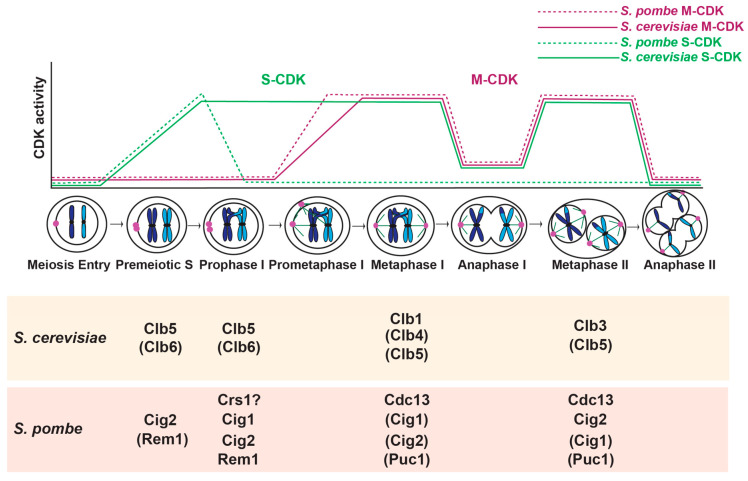
Oscillation of cyclin-dependent kinase (CDK) activity and cyclins present in each stage of meiosis. In budding yeast, S phase CDK (S-CDK) activity peaks during premeiotic S phase and remains high until anaphase I. Following degradation of S phase cyclin, S-CDK activity increases again during metaphase II, followed by a decrease in anaphase II. In fission yeast, S-CDK activity peaks during premeiotic S phase, then declines following a decrease in Cig2 protein. In both budding yeast and fission yeast, M-CDK activity is low until entry into the nuclear divisions, during which M phase CDK (M-CDK) activity peaks at metaphase I and metaphase II. There is a highly regulated partial drop in M-CDK activity between meiosis I and meiosis II. For both yeast species, cyclins are listed under the stage in which they act. Cyclins in parenthesis have more minor or undetermined roles during these stages. Question mark after a cyclin indicates conflicting data about that cyclin’s role in the corresponding stage.

Budding and fission yeast have also been instrumental in revealing basic principles of meiotic regulation. Starvation of key nutrients induces meiosis in both organisms [38]. Cells undergo one round of DNA replication followed by a prolonged prophase I, in which homologous chromosomes pair, synapse (in budding yeast) and recombine (Figure 1) [39,40]. In meiosis I, the paired homologous chromosomes attach to spindle microtubules and segregate. In meiosis II, sister chromatid kinetochores attach to spindle microtubules and separate. The four haploid meiotic products are packaged into spores and germinate when nutrients return. 

CDK regulates many meiotic events, with important roles in S and M phases, similar to the functions of CDK in the mitotic cell cycle. In addition, CDK regulates meiosis-specific events to ensure that homologs make interactions in prophase I, that DNA is not re-replicated in between the two meiotic divisions, and that cells undergo two and only two meiotic divisions. In this review, we highlight the complex regulation of Cdk1 and cyclins in meiosis in budding and fission yeast. We discuss how the expression of cyclin genes is regulated, how post-translational mechanisms of cyclins and Cdk1 affect activity, and how degradation of the cyclins and other cell cycle proteins is regulated. Finally, we compare the regulation between the two model organisms to highlight the differences in regulation that accomplish a similar outcome. 

## 2. Overview of *S. cerevisiae* CDK Activity

In budding yeast, progression through meiosis is governed by the oscillatory activity of CDK and a CDK-related kinase Ime2. Budding yeast have six conserved CDKs, but only one, Cdk1 (or Cdc28), is required for mitotic and meiotic cell cycle control [28]. One other CDK, Pho85, has minor roles in G1 and S phase and may function to fine-tune cell cycle regulation, or may have more important functions under specific environmental conditions [41,42,43]. Whether Pho85 has a similar role in meiosis has not been investigated. There are nine cyclins that bind and activate Cdk1 in the mitotic cell cycle, the G1 cyclins Cln1, Cln2, and Cln3, and six B-type cyclins Clb1-6. In meiosis, the G1 cyclins are not expressed. Instead, Ime2 is required for the meiotic G1/S transition [44,45]. Cdk1 bound to Clb5 and Clb6 are important for DNA replication in premeiotic S phase [44,46] (Figure 1). Ime2 and CDK bound to B-type cyclins regulate the meiotic divisions [47,48]. The major mitotic cyclin, Clb2, is not expressed in meiosis [49,50]. Instead, Clb1 and Clb3 are considered the major meiosis I and meiosis II cyclins, respectively [51]. 

Full Cdk1 activity requires the binding of cyclins as well as the phosphorylation of the T-loop by CDK activating kinase Cak1 [28]. The non-phosphorylated T-loop blocks the active site of Cdk1, and its phosphorylation by Cak1 is thought to reposition the T-loop to allow access to the substrate-binding domain and to increase the affinity for cyclins [52,53,54,55,56,57,58]. Cak1 also phosphorylates the Ime2 activation loop, which results in Ime2 autophosphorylation and activation [59]. Although Ime2 is related to CDKs, it does not interact with cyclins to function [60].

CDK activity is downregulated at meiotic entry and during anaphase I and anaphase II. Different mechanisms are used to attenuate CDK activity. The CDK inhibitor Sic1 keeps CDK activity low as cells enter meiosis [44,47]. Post-translational modifications of the cyclins, such as phosphorylation, can decrease CDK activity at anaphase I [51,61,62,63]. Also, some cyclins are degraded in prophase I, anaphase I, and anaphase II [64,65,66]. The APC/C binds to either the co-activator Cdc20, or the meiosis-specific co-activator Ama1 to ubiquitinate and target the cyclins for proteasomal degradation (Table 1) [64,65,66,67,68]. 

### 2.1. Entry into Meiosis and Premeiotic DNA Replication

In budding yeast, meiosis is orchestrated through a series of transcriptional cascades. A transcriptional activator, Ime1, is induced in diploid cells through the integration of a number of external signals including nitrogen levels, carbon source, external pH, and presence of both mating type loci [38,69,70,71,72,73]. Interestingly, G1-CDK activity prevents the expression of Ime1, as does the presence of glucose and nitrogen [73,74]. In the lab, the induction of Ime1 typically occurs through nitrogen depletion and the presence of a nonfermentable carbon source such as acetate [71]. The activity of Ime1 initiates the first transcriptional wave of early meiotic genes needed for entry into meiosis, premeiotic S phase, prophase I, and prophase I exit [45,75,76,77,78,79]. One of the genes targeted by Ime1 is *IME2*, whose production is important for many events throughout meiosis [70,75,76,80].

In meiosis, G1-CDK is not active [74]. Instead, Ime2 performs many functions for the initiation of meiosis. For example, Ime2 is needed for the rapid upregulation of many early meiotic genes and for the timely entry into premeiotic S phase [80,81,82,83,84]. A comparison of RNA expression between wild type and *ime2Δ* cells shows that the expression of many cell cycle genes is decreased or delayed in *ime2Δ* cells, especially those with consensus SCB (Swi4 cell cycle box) or MCB (MluI cell cycle box) elements in their promoters [82]. In the mitotic cell cycle, the expression of genes with these promoter elements is normally regulated by G1-CDK. In addition, Ime2 is needed for the timely activation of S-CDK (Cdk1 bound to Clb5, or Clb6) [44,46,47]. In contrast to the mitotic cell cycle in which the CDK inhibitor Sic1 is phosphorylated and targeted for proteasomal degradation by G1-CDK, in meiosis, Ime2 activity results in Sic1 phosphorylation, targeting it for proteasomal degradation [7,44,46,47,74,82,85,86,87,88,89,90]. However, Sic1 is eventually degraded after a long delay in *ime2Δ* cells, suggesting redundant mechanisms that target Sic1 for degradation in meiosis [82].

Once Sic1 is degraded, S-CDK activity promotes premeiotic DNA replication [44,46,47]. Drug inhibition of CDK or deletion of both S phase cyclins Clb5 and Clb6 resulted in cells that were unable to replicate their DNA, demonstrating that S-CDK is essential for DNA replication. Clb5 is thought to be the more dominant cyclin for S-CDK activity. Cells lacking Clb5 are delayed in DNA replication and arrest in either prophase I or in meiosis I, while cells lacking Clb6 undergo a normal meiosis [44]. How S-CDK regulates premeiotic DNA replication has not been fully characterized, but we assume that it phosphorylates DNA replication proteins, as it does in the mitotic cell cycle. In addition, another important kinase DDK (Dbf4-dependent kinase), which consists of the Cdc7 catalytic subunit and the Dbf4 regulatory subunit is also important for DNA replication in both the mitotic cell cycle and meiosis [91,92,93,94]. 

### 2.2. Prophase I

After cells replicate their DNA, they enter an extended prophase I in which chromosomes pair, undergo programmed DNA double strand breaks (DSBs), assemble synaptonemal complex, and repair the DSBs off the homolog for crossover recombination [40]. During this process, chromosomes are undergoing rapid telomere-led movements, in which telomeres first cluster together at the nuclear periphery in a “bouquet” configuration and attach to the cytoplasmic cytoskeleton through a protein complex embedded in the nuclear membrane. In budding yeast, the movements are dependent on the actin cytoskeleton and are thought to resolve heterologous interactions and chromosome entanglements [40,95,96,97,98,99,100,101].

S-CDK helps orchestrate many events throughout prophase I with roles in DSB formation and processing and synaptonemal complex assembly [102,103,104,105]. S-CDK and DDK phosphorylate Mer2, an accessory factor for the Spo11 enzyme that makes the DSBs, and this phosphorylation mediates an interaction between Mer2 and other proteins involved in DSB formation [103,105,106,107,108,109]. The timing of Mer2 phosphorylation is coordinated with replication by the recruitment of DDK to the replisome [108,109]. S-CDK also phosphorylates Sae2, a DSB repair protein needed for the removal of the covalent attachment between Spo11 and the DNA ends to allow further processing of DSBs [110]. In addition, CDK is necessary for normal synaptonemal complex formation and may phosphorylate several synaptonemal complex components [104].

During prophase I, it is imperative that M-CDK is not active. Over-expression of the genes encoding the *CLB1* and *CLB3* cyclins during prophase I causes premature spindle assembly and premature interactions between kinetochores and microtubules [111]. When these cells undergo anaphase I, sister chromatids separate instead of homologous chromosomes. Interestingly, premature expression of *CLB4* results in premature spindle formation, but kinetochores do not engage with microtubules. When cells that prematurely expressed *CLB4* are released from the prophase I arrest, they segregate homologous chromosomes. These results suggest that premature kinetochore-microtubule interactions can lead to a disruption in the meiosis I chromosome segregation pattern and that the activity of CDK, especially when bound to Clb1 or Clb3, needs to be tightly regulated.

Multiple mechanisms restrict M-CDK activity in prophase I. First, the APC/C, bound to the meiosis-specific co-activator Ama1 targets M phase regulators and M phase cyclins for proteasomal degradation during prophase I (Table 1) [66]. One of the M phase regulators that is targeted by APC/C-Ama1 is Ndd1, a transcriptional activator for the M phase cyclins in mitosis. By targeting both Ndd1 and the M phase cyclins for degradation, Ama1 prevents premature activity of M-CDK. Second, transcription of the M phase cyclins depends on Ndt80, which induces gene expression at the end of the pachytene stage of prophase I [76,112]. Third, the meiotic recombination checkpoint, which delays cells in prophase I for additional time to repair DSBs, either directly inhibits M-CDK activity or inhibits the transcription of the M phase cyclins [113,114,115,116]. The recombination checkpoint is normally always active in meiosis as cells are repairing DSBs, and the delay can be extended if DSBs are not repaired. DSBs mediate checkpoint signaling by activating either Tel1 (ATM homolog in mammals) or Mec1 (ATR homolog in mammals), depending on the processing status of the lesion [117]. Mec1 and Tel1 activate a meiosis-specific effector kinase Mek1 that helps to inhibit meiotic progression to promote repair [118,119,120,121,122,123]. Mek1 phosphorylates and inhibits the middle meiosis transcription factor Ndt80, which prevents transcription of the M phase cyclins to block entry into the meiotic divisions [114]. The recombination checkpoint is also signaled through the activation of the Swe1 kinase (Wee1 in fission yeast and mammals), which puts an inhibitory phosphorylation on CDK [113]. 

Transcription of *NDT80* is tightly regulated to prevent premature expression of middle meiosis genes. Although Ime1 induces the transcription of *NDT80*, expression is delayed with respect to other Ime1-induced genes because the Sum1 repressor binds to the *NDT80* promoter [112,124,125]. Sum1 is removed from the promoter once phosphorylated by Ime2, CDK, and DDK [126,127,128]. Therefore, Ndt80 is only expressed once Ime2, also induced by Ime1, is active. Ime2 also phosphorylates and activates Ndt80 [47]. The small amount of Ndt80 from Ime1 induction is held inactive until the recombination checkpoint is satisfied [129]. Once DSBs are repaired and the checkpoint is no longer active, Ndt80 can then bind its own promoter to give a high induction of *NDT80* transcription [130]. Ndt80 induces the expression of greater than 300 target genes, many of which are involved in the completion of meiotic recombination, the disassembly of the synaptonemal complex, the meiotic divisions, and spore formation, ultimately resulting in prophase I exit [112,129,131]. 

### 2.3. The Meiotic Divisions

CDK activity in conjunction with the B-type cyclins Clb1, Clb3, and Clb4 promote progression through the meiotic divisions [47,49,50] (Figure 1). *CLB2*, which encodes the major mitotic cyclin, is not expressed in meiosis [49,50]. Cells with single deletions of *CLB1*, *CLB3*, or *CLB4* undergo meiosis somewhat normally, except that loss of *CLB1* causes a reduction in four-spored asci and an increase in two-spored asci [50]. Clb1 seems to be the most important cyclin for meiosis, as combined loss *clb1Δ clb3Δ* and *clb1Δ clb4Δ* mutants have a much higher frequency of dyads than *clb3Δ clb4Δ* mutants. The dyads produced from *clb1Δ clb3Δ* and *clb1Δ clb4Δ* cells are viable and diploid. Examination of marker segregation suggests that the cells undergo meiosis I but not meiosis II. Overall, the results demonstrate that the M phase cyclins can compensate for one another, but that Clb1 may have a more important role than other M phase cyclins.

Each B-type cyclin has a unique regulation [47,49,50,51,61]. A previous study characterized the regulation of B-type cyclin mRNA, protein, and CDK activity [51]. They find that Clb5 protein and CDK-Clb5 activity both increase at metaphase I and metaphase II, and decline in anaphase I and anaphase II. Clb4 protein accumulates in meiosis I and stays present throughout both meiotic divisions. Although CDK-Clb4 is active in meiosis I, its activity declines in meiosis II, suggesting that post-translational mechanisms regulate CDK-Clb4 activity. Clb1 protein levels increase in meiosis I and remain high until meiosis II exit. However, a different study finds that the levels of Clb1 in the nucleus decrease in anaphase I and then return in metaphase II [62]. In addition, CDK-Clb1 is only active in meiosis I, again suggesting regulation through post-translational mechanisms [51]. Although *CLB3* is transcribed by Ndt80 and the RNA is present in meiosis I, *CLB3* mRNA is not translated until meiosis II. *CLB3* mRNA is not translated because it binds a translational repressor until meiosis II [132]. Forced production of Clb3 in meiosis I results in the loss of the meiosis I chromosome segregation pattern: sister chromatids separated instead of homologous chromosomes [51,111]. These results suggest that Clb1 serves as the major meiosis I cyclin and Clb3 serves as the major meiosis II cyclin. Further work is needed to understand the roles of Clb4 and Clb5 in the meiotic divisions. The finding that meiosis can occur mostly normally without Clb1 or Clb3 suggests that the other cyclins likely have overlapping roles in meiotic progression [50].

**Figure 2:**
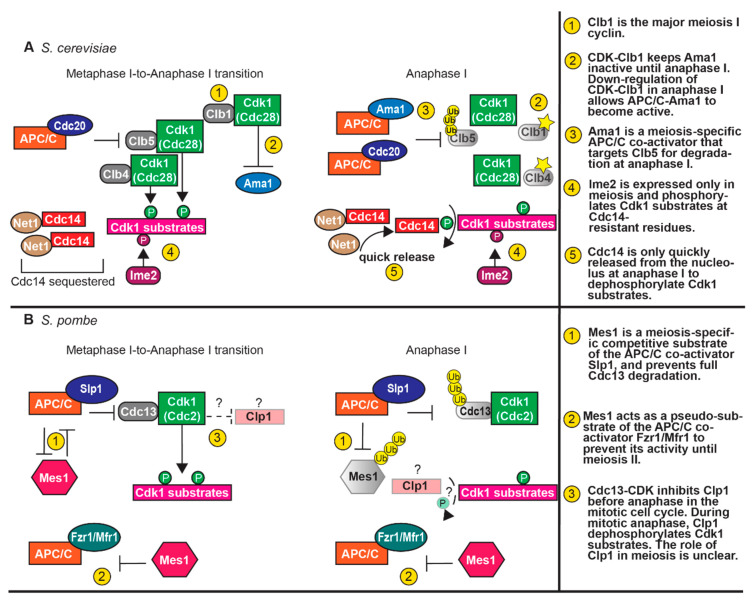
Regulation of CDK activity in meiosis I. (**A**) At the metaphase I-to-anaphase. I transition in budding yeast meiosis Cdc14 is held inactive by Net1. APC/C-Cdc20 targets Clb5 for degradation while the APC/C co-activator Ama1 is inhibited by CDK-Clb1. Ime2 phosphorylates a subset of Cdk1 substrates. At anaphase I, Ama1 becomes active and APC/C-Ama1 targets Clb5 for further degradation. Clb1 and Clb4 are likely post-translationally modified (denoted with star) to downregulate CDK activity. Cdc14 activation is promoted by only the FEAR pathway, causing a partial but not full dephosphorylation of CDK substrates. Cdc14 reverses CDK, but not Ime2, phosphorylation on substrates. Combined, this regulation leads to a decline in CDK activity. (**B**) In fission yeast meiosis, Mes1 inhibits the APC/C. Mes1 is a competitive substrate of the APC/C co-activator Slp1 (Cdc20) and a pseudo-substrate of the co-activator Fzr1/Mfr1. Mes1 prevents full degradation of Cdc13 during meiosis I, thus allowing for retention of some CDK activity. Progression into anaphase I requires APC/C ubiquitination and proteasomal degradation of Mes1. Mes1 inhibits Fzr1/Mfr1 until meiosis II. The role of Clp1 (Cdc14) in meiosis is unclear, as deletion of clp1 has no effect on the timing of meiosis, but has a minor effect on the formation of four-spore asci.

The transition between meiosis I and meiosis II requires unique regulation (Figure 2). As cells undergo anaphase I, the APC/C bound to the co-activator Cdc20 becomes active and ubiquitinates securin, targeting it for proteasomal degradation [67]. Securin degradation releases separase, which cleaves cohesin along the chromosome arms for chiasma release, allowing homologous chromosome segregation. The pericentromeric cohesin is protected from cleavage by shugoshin (Sgo1) so that sister chromatids stay together until meiosis II [133,134]. The levels of Clb5 also decline in anaphase I, likely due to targeting by APC/C-Cdc20 [51,135] (Table 1). With the post-translational downregulation of Cdk-Clb1 activity, APC/C-Ama1 becomes active and also targets Clb5 and securin for degradation [65]. Thus, there are two pathways of APC/C-mediated degradation to promote timely degradation of securin and cyclins during anaphase I.

With the degradation or other post-translational regulation of the cyclins, CDK activity decreases and the Cdc14 phosphatase is released from the nucleolus to reverse CDK phosphorylations. Both the low level of CDK activity and the dephosphorylation of substrates is required for meiosis I spindle disassembly [62,63]. However, origin licensing, which normally occurs with low CDK activity, is suppressed after meiosis I to prevent DNA re-replication. Budding yeast likely undertake two mechanisms to prevent DNA re-replication. First, CDK activity is not fully downregulated and Cdc14 nucleolar release is not as extensive in meiosis as in mitosis [62,63,136]. In mitosis, there are two pathways that lead to full Cdc14 release, FEAR (Cdc14 early anaphase release) and MEN (mitotic exit network) [33,137,138,139]. In meiosis, Cdc14 release primarily occurs through FEAR and not MEN, suggesting that the release may not be as prolonged [63,136]. Second, CDK and Ime2 phosphorylate many of the same substrates but at different consensus sequences, including the CDK inhibitor Sic1, and pre-Replication Complex (pre-RC) components Cdc6, Mcm6, and Orc6 [140,141]. The Ime2 phosphorylations are thought to have the same functional consequence as the CDK phosphorylations, except that they are resistant to the Cdc14 phosphatase. The pre-RC substrates do not assemble due to the activity of both CDK and Ime2 [141]. Furthermore, Ime2 was shown to be sufficient to block DNA re-replication [140]. Also, some *ime2Δ* cells undergo a second round of DNA replication in late meiosis, after a prolonged delay [81,142]. Therefore, by having two kinases that phosphorylate many of the same substrates and some non-overlapping substrates, cells can transition from meiosis I to meiosis II without DNA re-replication. 

In meiosis II, a number of mRNAs, including *CLB3* mRNA, are translated for normal meiosis II regulation, meiotic exit, and spore formation. The Rim4 translational repressor forms amyloid-like aggregates and binds to the 5′UTR of *CLB3* and other mRNAs until meiosis II [132,143]. In meiosis II, Ime2 heavily phosphorylates the intrinsically disordered region of Rim4, leading to some reversal of the amyloid-like properties of Rim4 [144]. The phosphorylation of Rim4 likely leads to its autophagic and/or proteasomal degradation [144,145]. 

Rim4 is a substrate of autophagy, and with inhibition of autophagy, Rim4 persists and Clb3 protein is not present [145,146]. Interestingly, inhibition of autophagy blocks meiotic exit and the cells continue to undergo rounds of spindle formation, spindle elongation, and chromosome segregation. Rim4 also binds mRNA of the meiosis-specific co-factor of the APC/C, Ama1 [143]. Ama1 substrates are not degraded with inhibition of autophagy, suggesting that Ama1 is not present in high enough levels for APC/C-Ama1 activity [145]. Forced expression of *CLB3* or *AMA1* in meiosis II without their normal 5′UTR prevents the additional rounds of spindle formation and chromosome segregation. Therefore, autophagy has an important role in the regulation of meiotic exit and in preventing additional rounds of chromosome segregation by degrading Rim4 and allowing the translation of several mRNAs in meiosis II. 

One of the key steps for meiotic exit is the inactivation of M-CDK activity [64]. The M phase cyclins are targeted for proteasomal degradation, first by APC/C-Cdc20, whose activation in meiosis II requires the activity of the casein kinase Hrr25. Proteasomal degradation of the cyclins results in decreased CDK activity, which allows APC/C-Ama1 to become active. APC/C-Ama1 targets additional substrates for degradation, such as Ndt80, which prevents further transcription of the genes encoding the B-type cyclins and other M phase regulators. With the downregulation of M-CDK activity and the activation of APC/C-Ama1, the spindle disassembles and Cdc14 is released from the nucleolus for reversal of CDK substrate phosphorylations. Thus, cells exit meiosis and initiate the sporulation program. 

## 3. Overview of *S. pombe* CDK Activity

Meiosis in fission yeast is also governed by the oscillatory activity of CDK. Fission yeast encode six CDKs and two CDK activating kinases (CAKs) [147]. Only one CDK, Cdc2 (or Cdk1), is essential for mitotic and meiotic cell cycle regulation [148,149]. Although, Pef1 (similar to Pho85 in budding yeast), when bound to cyclin Pas1, promotes cell cycle start [150]. It is not known if Pef1 also has a role in meiosis. There are four cyclins that bind and activate Cdc2 in both the mitotic cell cycle and meiosis [151,152,153,154,155,156,157,158]. In addition, there are two meiosis-specific cyclins, Rem1 and Crs1 [159,160,161] (Figure 1). The cyclins Cig1, Cig2, and Puc1 are considered the G1/S cyclins with roles in entrance into S phase and DNA replication during vegetative growth [151,152,156,157,162]. In meiosis, among the G1/S cyclins, Cig2 has the most important role in DNA replication [163,164]. During prophase I, Cig1, Cig2, Crs1, and Rem1 contribute to programmed DSB formation, assembly of chromosomal architecture elements, or meiotic recombination [159,164,165,166]. The cyclin Cdc13 regulates chromosome segregation during mitosis and meiosis [148,163,167,168]. 

Full CDK activity requires both cyclin binding and the phosphorylation of the activation domain by CAK. Fission yeast have two CAKs, the Mcs6/Mcs2/Pmh1 complex and Csk1, an ortholog of budding yeast Cak1 [147,169,170,171,172,173,174,175,176]. Although *mcs6* is an essential gene and *csk1* is not essential, the Mcs6 complex and Csk1 are thought to have redundant roles in phosphorylating and activating Cdc2 [174,175]. In addition, Csk1 also phosphorylates and activates Mcs6, acting as a CAK activating kinase [177]. Although it is assumed that CAK activation of Cdc2 is also required for meiosis, roles of the Mcs6 complex and Csk1 have not been analyzed in meiosis. However, a recent study identified *csk1* in a genome-scale screen for genes important for meiosis [178]. Further work is needed to determine the role of Csk1 in meiotic regulation and spore formation, especially given the similarity of Csk1 to Cak1, which has additional roles in meiosis in budding yeast [59,176]. 

Oscillations in CDK activity occur through several regulatory mechanisms in meiosis. First, the cyclin-dependent kinase inhibitor Rum1 is needed for cells to arrest in G1 for mating and meiotic initiation, but Rum1 must be degraded prior to premeiotic S phase [179]. Second, M-CDK activity is regulated by the Wee1 kinase and the Cdc25 phosphatase during S phase and prophase I [163,168,180]. Wee1 phosphorylates tyrosine-15 on Cdc2 to decrease Cdc2-Cdc13 activity prior to entrance into meiosis I [180]. Cdc25 removes the inhibitory phosphorylation for a burst of CDK activity as cells enter meiosis I [148,163,168]. Third, many of the cyclins are regulated by meiosis-specific transcriptional and post-transcriptional mechanisms to allow an increase in cyclin levels at specific stages of meiosis [148,160,163,166,181,182,183]. Fourth, the cyclins are ubiquitinated and targeted for proteasomal degradation in anaphase I and anaphase II by the APC/C ubiquitin ligase. In meiosis, the APC/C mainly uses two co-activators, Slp1 (Cdc20 in budding yeast) and a meiosis-specific co-activator Mfr1/Fzr1 [184,185,186,187] (Table 1). 

### 3.1. Entry into Meiosis and Premeiotic DNA Replication

Fission yeast typically live as haploids, but cells of opposite mating type conjugate when starved of nitrogen [188,189]. The diploid zygote then undergoes meiosis and spore formation. In the lab, this method of sporulation is not very synchronous due to the conjugation step [39,190]. Often, a method is used, in which a diploid is induced to initiate meiosis through the downregulation of Pat1 activity, an inhibitor of meiosis (see below); however, some differences from zygotic meiosis can be observed [39]. 

Meiosis is initiated through the induction of successive waves of transcription. The expression of the transcripts at the mating loci induce the expression of *mei3*, which encodes an inhibitor of Pat1 protein kinase [191,192]. In vegetative cells, Pat1 blocks meiosis by phosphorylating the substrates Ste11, a transcriptional activator, and Mei2, an RRM-type RNA-binding protein under Ste11-transcriptional control [193,194]. Because Mei3 inhibits Pat1 in meiosis, Pat1 cannot promote Ste11 nuclear exclusion or target Mei2 for degradation. Thus, Ste11 induces the transcription of early meiotic genes, and levels of Mei2 accumulate [194,195]. Mei2 binds Mmi1 to inhibit DSR-Mmi1 (Determinant of Selective Removal-Mmi1), an RNA degradation system that selectively removes sporulation-specific transcripts [196,197]. Mei2 prevents Mmi1 from binding elements within the 3’UTR of meiotic transcripts and recruits the exosome to degrade them [196,198,199]. Therefore, the activation of both Ste11 and Mei2 allows hundreds of genes to be transcribed and meiotic mRNAs to be translated.

Two mRNAs regulated by Mei2 include *crs1*, which encodes a meiosis-specific cyclin and *mei4*, which encodes a transcription factor important for the expression of a large number of middle meiosis genes [160,163,181,200,201,202]. The transcription of both *crs1* and *mei4* occurs in vegetative and meiotic cells, but the RNA accumulates only upon Mmi1 inhibition in meiosis [181,196,202]. In addition, *csr1*, like many other meiotic transcripts in fission yeast, undergoes meiosis-induced splicing to restrict expression to meiosis [160,203]. Mei4 regulates expression of the genes that encode the cyclins *rem1*, and *cig2*, and the CDK regulators *wee1* and *cdc25* [159,163,166,204]. Interestingly, Mei4 also regulates the splicing of *rem1*, likely by recruiting components of the spliceosome [159,166]. Therefore, Mei2′s role in blocking RNA degradation of *crs1* and *mei4*, along with other transcripts, promotes many events throughout early meiosis and the meiotic divisions.

CDK activity is required for DNA replication in premeiotic S phase, likely due to its role in phosphorylating key proteins that regulate replication, as it does in the mitotic cell cycle [148,205]. *cig2* is transcribed just before premeiotic DNA replication, and the protein is present during S phase and then declines shortly afterwards [163]. In contrast, *cig1* is transcribed, and the protein present, just after premeiotic S phase and is not thought to have an important role in DNA replication [163,184]. The Puc1 cyclin also does not have a predominant role in premeiotic DNA replication [164]. Therefore, Cig2 bound to Cdc2 has a central role in DNA replication (Figure 1). However, cells with a deletion of *cig2* or deletions of both *cig1* and *cig2* only have a 30-minute delay in DNA replication. A triple deletion strain, *cig1Δ cig2Δ puc1Δ*, displays only minor additive roles in the timing of onset of premeiotic DNA replication. Therefore, the G1/S cyclins are dispensable for premeiotic DNA replication but make the process more efficient. 

The meiosis-specific cyclin Rem1 compensates for the loss of Cig2 to allow premeiotic DNA replication after a short delay [159]. Loss of both *cig2* and *rem1* results in cells that are blocked before premeiotic DNA replication. Normally, Rem1 is not present during premeiotic S phase; *rem1* is expressed prior to premeiotic DNA replication but the transcript is not spliced until just before the onset of meiosis I. However, *rem1* mRNA is spliced several hours earlier in *cig2Δ* cells, suggesting that CDK-Cig2 activity blocks *rem1* splicing. 

The M-phase cyclin Cdc13, and its associated kinase activity, is also present during premeiotic S phase and increases to high levels through meiosis II [163,168]. However, Cdc13 is not required for premeiotic S phase under normal circumstances and cannot compensate for the loss of all other cyclins in premeiotic S phase. Surprisingly, expression of a Cdc13-Cdc2 fusion protein is sufficient for DNA replication in the absence of all other cyclins [164]. These results suggest that Cdc2-Cdc13 can indeed support premeiotic DNA replication but is prevented through an unknown mechanism from activating CDK during normal premeiotic S phase. 

After cells complete S phase, the activity of CDK is reduced [163]. Cdc2 is phosphorylated at tyrosine-15, an inhibitory phosphorylation put on by Wee1 kinase [20,21,22,163,180,206,207]. Due to the inhibitory phosphorylation, the activity of Cdc2 bound to M phase cyclin Cdc13 is low, but not absent, until entry into meiosis I when the inhibitory phosphorylation is removed [163]. 

### 3.2. Prophase I

During prophase I, fission yeast undergo characteristic nuclear movements to help facilitate homologous chromosome pairing and recombination. Starting in premeiotic S phase, telomeres cluster together at the nuclear membrane, just underneath the SPB, and form a structure known as the telomere bouquet [208,209,210,211,212,213]. The bouquet is connected to the SPB. Although the telomere bouquet is a conserved structure found in many organisms, fission yeast have an especially remarkable bouquet stage [40,214]. Following bouquet formation, the nucleus oscillates due to microtubule and dynein-dependent drastic movements, led by the SPB inserted in the nuclear membrane [190]. This period is defined as the ‘horsetail stage’. The telomere bouquet and horsetail movements are important for a number of events including promotion of homolog interactions, disruption of ectopic interactions, meiotic recombination, spindle formation, and centromere assembly [215,216,217,218,219,220,221]. 

CDK activity regulates multiple events in prophase I, including DSB formation, recombination, and maturation of linear elements, which are protein complexes structurally related to elements within the synaptonemal complex in other eukaryotic organisms [165,222,223,224,225]. Along with Cig1 and Cig2, the meiosis-specific Crs1 cyclin is present in prophase I. *crs1* has four introns that are spliced starting in premeiotic S phase, and the spliced messages increased by prophase I [160]. Rem1 also has a role in recombination, such that levels of gene conversion, but not crossovers, are reduced in its absence [159]. Interestingly, Rem1 is not spliced until several hours after *crs1* [159,160,166]. In prophase I, the unspliced version of *rem1* is translated into a truncated protein that lacks a cyclin box. The shorter Rem1 is needed for normal levels of gene conversion, suggesting that Rem1’s role in recombination does not depend on binding Cdc2 [166]. Therefore, Rem1 has CDK-independent functions in prophase I, and Cig1, Cig2, and Crs1 would be the cyclins that function with Cdc2 to regulate events in prophase I.

As in budding yeast, DSBs are induced by the Spo11 homolog Rec12 bound to accessory proteins [226,227,228]. Inhibition of an analog-sensitive allele of Cdc2 (Cdc2-asM17) resulted in a great decrease in DSB formation [165]. Deletions of the cyclin genes *cig1*, *cig2*, and *crs1* individually cause a reduction in DSB formation at a strong natural hotspot, with the *cig1Δ crs1Δ* double mutant displaying the greatest reduction. Linear elements are required for normal double strand break formation at hotspots [161,222,224,229]. *cig1Δ crs1Δ* double mutants accumulate linear element protein Rec25 with similar timing as control cells but few cells display mature linear elements [165]. Similar results are found in *cdc2-asM17* cells, even without addition of the analog, and the defects are enhanced upon analog addition. These results suggest that CDK activity is needed for linear element maturation and normal DSB formation. 

Cig1, Cig2, and Crs1 also contribute to recombination. Deletion of *cig1* or *cig2* caused a reduction in gene conversion, but not a reduction in crossovers [165]. The *cig1Δ cig2Δ* double mutant did not have an additive effect. Loss of *crs1* resulted in a decrease in both gene conversion and crossovers. The *cig1Δ crs1Δ* double mutant had a similar defect as the *crs1Δ* single mutant. In another study, loss of *crs1* shows no defect in recombination [164]. It is currently unclear why the two studies gave conflicting results. However, the summary of results suggests that CDK activity, with Cig1, Cig2, and possibly Crs1, regulates DSB formation, linear element maturation, and recombination, but the substrates are currently not known. Since these events are linked, with lower levels of mature linear elements affecting DSB formation and recombination, a small number of substrates may regulate all three events. [165,222,223,224,230,231,232,233,234]. Future studies are needed to identify the substrates that regulate these events.

The completion of recombination is monitored by the recombination checkpoint, which delays prophase I progression by downregulating CDK activity in the presence of unrepaired DNA breaks. DSBs are sensed by Rad3 (ATR) and Tel1 (ATM), which phosphorylate and activate the downstream meiosis-specific effector kinase Mek1 [235,236,237]. Mek1 excludes Cdc25 from the nucleus to prevent the removal of the inhibitory phosphorylation of Cdc2, resulting in an inhibition of CDK activity [238]. Chk1 acts downstream of Rad3 and Tel1 in the mitotic DNA damage checkpoint to phosphorylate both Cdc25 and Wee1 [239,240]. However, although Chk1 is predicted to play a role in the recombination checkpoint, Chk1 is not active until meiosis II [236]. Activation of the recombination checkpoint, by mutating proteins involved in DNA repair, results in an extended meiotic prophase beyond the completion of horsetail movements [241]. 

### 3.3. The Meiotic Divisions

The normal transition from prophase I to the entrance into the first meiotic division is also controlled by Wee1 and Cdc25 regulation of CDK. Cells enter prophase I with low CDK activity due to Wee1 inhibitory phosphorylation on Cdc2 [163,168,180]. As cells progress through prophase I, and DNA breaks are repaired, the levels of phosphorylated Cdc2 decreases due to the activity of the Cdc25 phosphatase, with a corresponding increase in CDK activity [163,168]. The Mei4 middle meiosis transcription factor induces *cdc25* and inhibits *wee1* by binding a region upstream of the *wee1* open reading frame [148,204,242]. This transcriptional regulation results in an increase in Cdc25 and a decrease in Wee1 as cells progress through prophase I, allowing a rapid induction of CDK activity and the entrance into nuclear divisions. 

The M phase cyclin Cdc13 is essential for the meiotic divisions. In the absence of Cdc13, cells undergo premeiotic DNA replication, but fail to undergo the meiotic divisions [148,164,205]. Some of the cells even re-replicate their DNA [164]. Cdc13 protein is present in S phase and then increases in the meiotic divisions [163,168]. The increase in protein levels is due to meiosis-specific transcriptional and post-transcriptional regulation of *cdc13*. Instead of producing a single 2.5kb transcript, as found in mitosis, both a 2.5kb and a 2.2kb *cdc13* transcript is produced in meiosis, with a different length in the 3’UTR [148,163]. *cdc13* transcription initiates in premeiotic S phase and rises to a higher level in meiosis I and meiosis II [163]. In the absence of the transcriptional activator Mei4, the smaller transcript did not rise to high levels in meiosis I and meiosis II, as it does in wildtype cells [148,163]. The transcript is also regulated by the Spo5 RNA-binding protein [182]. Spo5 protein is present in prophase I and then disappears as cells enter meiosis II, similar to the timings when Cdc13 levels increase [182,183]. In the absence of *spo5*, cells had lower levels of Cdc13 in meiosis II and did not complete meiosis II, suggesting that Spo5 has an important role in allowing the proper timing of the production of Cdc13. The meiosis II defect is partially suppressed by overexpression of Cdc13, suggesting that lower levels of Cdc2-Cdc13 activity prevents *spo5Δ* cells from finishing meiosis II [182,183]. The transcriptional and post-transcriptional regulation of *cdc13* is thought to allow an increase in Cdc13 protein in meiosis II, after degradation of Cdc13 in anaphase I, such that adequate Cdc13 protein is present for meiosis II.

With endogenous expression, Cdc13 is not sufficient for the production of viable gametes. Cells with deletions of all other cyclins besides Cdc13 (*cig1Δ cig2Δ puc1Δ rem1Δ crs1Δ*) form mostly empty or one-spored asci [164]. However, if Cdc13 is fused to Cdc2, cells lacking the other cyclins undergo meiosis I, but arrest before meiosis II. A higher level of expression of Cdc2 fused to Cdc13 allows cells to go through both meiotic divisions, however, most of the spores are inviable. These results suggest that Cdc13 is sufficient for progression through both divisions when tethered to Cdc2 and expressed at a high level, but other cyclins are needed for spore viability, likely due to their important roles in recombination and other meiotic events. 

Cig2, Rem1, and Crs1 have more minor roles in the meiotic divisions. Although the main role of Cig2 is in premeiotic DNA replication, Cdc2-Cig2 has another peak of expression (Mei4-dependent) and activity at the end of meiosis I [163] (Figure 1). Most *cig2Δ* cells undergo meiosis normally, but some have a delay in meiosis II and form dyads, with two diploid spores [163,164]. The percentage of dyads increases in *cig1*Δ*cig2*Δ double mutants and *cig1Δ cig2Δ puc1Δ* triple mutants [160,164]. Rem1 and Crs1 are only important for zygotic meiosis, in which haploid cells mate before undergoing meiosis. Some *rem1Δ crs1Δ* cells underwent abnormal meiotic nuclear divisions due to a multipolar spindle. In conclusion, Cig1, Cig2, and Puc1 have some additional roles in progression through the meiotic divisions. 

As cells exit metaphase I, the APC/C becomes active to ubiquitinate securin and B-type cyclins, targeting them for proteasomal degradation. The degradation of securin releases separase, which cleaves cohesin and allows the separation of homologous chromosomes [243]. Cdc13 is also degraded, but a pool of Cdc13 remains for low CDK activity during the meiosis I-to-meiosis II transition (Figure 2) [244]. The decrease in some but not all CDK activity is important; CDK activity must be low enough to allow spindle disassembly and meiosis I exit, but high enough to prevent origin licensing and the re-replication of DNA. In fission yeast, an inhibitor of the APC/C, Mes1, prevents full Cdc13 degradation [244,245,246]. Mes1 is a pseudo-substrate of the APC/C co-activator Fzr1/Mfr1 and a competitive substrate of co-activator Slp1/Cdc20. As a pseudo-substrate, Mes1 binds and inactivates Fzr1 to prevent the premature activity of APC/C-Fzr1 in meiosis I. As a competitive substrate, Mes1 competes with Cdc13 for binding of APC/C-Slp1. Once bound, Mes1 is eventually ubiquitinated and released. By having Mes1 as a substrate, there is sufficient degradation of securin to facilitate chromosome segregation, but only partial degradation of Cdc13, thus reducing but not eliminating Cdc2-Cdc13 activity. 

There is a precise balance in maintaining the correct levels of Cdc13 between the meiotic divisions (Figure 2). In *mes1Δ* mutants, Cdc13 is completely degraded and cells cannot progress into meiosis II [187,244,245,246]. A loss of *fzr1* allows *mes1Δ* cells to undergo meiosis II, demonstrating that reducing APC/C activity is important for the transition to meiosis II [186,187,246]. However, too much of a reduction of APC/C activity can also be detrimental. A mutant of *slp1* (*slp1-B05*) that results in reduced APC/C-Slp1 activity, does not affect vegetative growth; but, in meiosis, metaphase I is substantially delayed and *slp1-B05* cells ultimately undergo only one meiotic division [186]. Deletion of both *mes1* and *fzr1* can rescue the second meiotic division. These results demonstrate the importance of Mes1 in allowing a decrease but not an elimination of Cdc2-Cdc13 activity. 

As cells enter meiosis II, APC/C-Slp1 and APC/C-Fzr1 activity declines and Mes1 is targeted for proteasomal degradation, possibly through other APC/C co-activators Fzr2 and Ste9, promoting full CDK activity and meiosis II spindle assembly [246]. In addition, the Cuf2 transcription factor induces expression of *fzr1* to increase Fzr1 levels in meiosis II [186]. The boost of APC/C-Fzr1 activity is required for normal meiotic exit and spore formation [184,185,246].

In anaphase II, both APC/C-Slp1 and APC/C-Fzr1 target securin and B-type cyclins for degradation [186]. Although *fzr1Δ* cells undergo meiosis I and meiosis II normally, they do not exit meiosis. Instead, they assemble abnormal spindles and undergo an additional round of chromosome segregation [186]. They also maintain levels of Cdc13 after meiosis II, unlike wildtype cells that degrade Cdc13 after meiosis II [184,186]. In mutants of *cuf2*, the transcription of *fzr1* is decreased by approximately 50% during the meiosis I-to-meiosis II transition [186]. Approximately 20% of *cuf2Δ* asci display additional spores, also due to an additional round of chromosome segregation after meiosis II. Cdc13 degradation is also aberrant in *cuf2Δ* cells. These results suggest that degradation of Cdc13 by APC/C-Fzr1 activity is important for the termination of meiosis after meiosis II. 

## 4. Comparisons of CDK Activity in Budding and Fission Yeast Meiosis 

CDK has many important roles in regulating both budding and fission yeast meiosis. Although many of the essential activities of CDK are similar between the two organisms, there are also interesting examples where regulation is quite different. These differences demonstrate how regulatory mechanisms have evolved between the two species but accomplish the same outcome. We highlight some of the similarities and differences in this section.

In both budding and fission yeast, meiosis initiates in diploid cells heterozygous for the mating loci as a response to starvation and is coupled to spore formation [38,69,70,71,72,73,188,190]. The starvation response signals the induction of transcriptional cascades that result in meiotic initiation [45,112,194,195]. As cells enter meiosis, CDK activity is low and inhibited by CDK inhibitors Sic1 and Rum1 in budding and fission yeast, respectively [44,179]. Budding yeast has a meiosis-specific CDK-like kinase called Ime2 that functions at multiple stages in meiosis, including entrance into premeiotic S phase and in meiosis II [44,45,47,48,144]. Ime2 targets Sic1, the CDK inhibitor, for degradation to allow activation of S-CDK [47]. In fission yeast, the CDK inhibitor Rum1 must also be degraded prior to premeiotic S phase, but it is currently unclear how Rum1 is targeted for degradation in meiosis [179]. Once active, S-CDK has an essential role in DNA replication in both yeasts, likely by phosphorylating the same substrates involved in mitotic DNA replication. 

In an extended prophase I, many meiosis-specific events occur, including DSB formation, homologous chromosome pairing, and recombination [40]. During this time, telomeres cluster into a bouquet structure and then make connections with proteins that pass through the nuclear envelope, linking telomeres to the cytoskeleton and facilitating rapid chromosome movements. Such telomere-led movements are thought to help facilitate homologous chromosome interactions, possibly by disrupting ectopic interactions and removing interlocks. Currently, it is unknown whether CDK regulates chromosome movements, but it is intriguing to speculate a role for CDK, given that other events in prophase I are known to require CDK. In mammals, CDK2 is involved in telomere attachment to the nuclear envelope during meiosis [247,248,249,250]. In budding yeast, CDK phosphorylates the Spo11 accessory protein Mer2 for formation of programmed DSBs, and CDK regulates Sae2 for break repair [103,105,110]. In fission yeast, CDK is also involved in DSB formation, but the substrates are unknown [165]. Cdc2 inhibition results in defective linear elements, possibly suggesting that CDK regulates DSB formation by regulating linear element assembly, which is required for normal levels of DSBs [222]. Future work is needed to determine whether there are multiple CDK substrates for DSB formation, linear element assembly, and recombination in fission yeast.

In both budding and fission yeast, a checkpoint in prophase I delays the cell cycle in the presence of unrepaired DSBs. One strategy for this checkpoint is to downregulate CDK activity by activating the Swe1/Wee1 kinase which places an inhibitory phosphorylation on CDK to block its activity. In budding yeast, Swe1 activation can result in checkpoint signaling, but is not thought to be a main mechanism of checkpoint regulation [113,117]. Instead, the checkpoint mainly functions to inhibit the Ndt80 transcription factor to prevent transcription of the M phase cyclins, thus preventing M-CDK activity [114,115,116]. In fission yeast, CDK inhibition is the main strategy for engaging the checkpoint and delaying cells in prophase I. Activation of the checkpoint prevents nuclear accumulation of the Cdc25 phosphatase, preventing CDK activation [237,238]. The difference in regulation between the two yeasts could be due to the timing of cyclin expression. In budding yeast, the M phase cyclins Clb1, Clb3, and Clb4 are not transcribed until cells are exiting prophase I, so inhibition of Ndt80, which is required for transcription of the cyclins and other meiotic regulators, keeps cells in prophase I [112]. Ndt80 also induces transcription of Polo kinase (Cdc5), which leads to disassembly of the synaptonemal complex [112,131]. Since the synaptonemal complex is needed for normal levels of homologous recombination, a checkpoint arrest through Ndt80 inhibition prevents premature synaptonemal complex disassembly, thus ensuring cells complete DSB repair through homologous recombination [118,131,251]. In fission yeast, the M phase cyclin Cdc13 is present in prophase I, and Wee1 inhibits Cdc2-Cdc13 activity throughout a normal prophase I [163,168]. Checkpoint activation prolongs CDK inhibition and extends the time in prophase I to allow DSB repair [168]. 

For budding and fission yeast cells to enter meiosis I, M-CDK activity increases to promote spindle assembly and chromosome attachment to spindle microtubules. In budding yeast, the M phase cyclins Clb1 and Clb3 are the main cyclins needed for meiosis I and meiosis II, respectively [51]. In fission yeast, Cdc13 regulates both meiotic divisions, but the levels increase in meiosis II [148,164,182,205,252]. In both yeasts, the cyclins that promote premeiotic DNA replication, Clb5 and Cig2 are also activated in the meiotic divisions [51,163]. However, the function of Clb5 in the meiotic divisions has not been delineated. Loss of *cig2* does affect meiosis II timing and spore formation [163,164]. Further experiments are needed to identify the specific roles for these cyclins in the meiotic divisions.

In anaphase I, CDK activity must be downregulated to allow spindle disassembly and meiosis I exit. In both budding and fission yeast, APC/C-Cdc20/Slp1 becomes active to target both securin and the cyclins for degradation at the metaphase I-to-anaphase I transition [67,187]. In budding yeast, some of the cyclins are post-translationally regulated for inactivation [51]. After inactivation of Cdk1-Clb1, which inhibits Ama1, APC/C-Ama1 becomes active and targets securin and cyclins for degradation [65]. The phosphatase Cdc14 is released from the nucleolus to dephosphorylate CDK substrates. Both the decline in CDK activity and the release of the Cdc14 phosphatase is needed for spindle disassembly [62,63]. In fission yeast, APC/C-Slp1 targets securin and Cdc13 for degradation at the metaphase I-to-anaphase I transition [187,246]. The Cdc14-like phosphatase Clp1 is not needed for normal meiotic timings or spindle disassembly, but may help with the fidelity of the process [35]. 

During the meiosis I-to-meiosis II transition, CDK activity must decrease for spindle disassembly, but some remaining CDK activity prevents re-licensing of origins. Budding and fission yeast have two different strategies to prevent complete loss of CDK activity, and consequentially, prevent full dephosphorylation of CDK substrates (Figure 2). In budding yeast, the Ime2 kinase remains active and phosphorylates some of the same substrates as CDK, including a component of the MCM helicase [140,141]. The Ime2 phosphorylations are not removed by the Cdc14 phosphatase, rendering Ime2 substrates resistant to Cdc14 dephosphorylation between meiosis I and meiosis II [140]. In addition, Cdc14 is only quickly released in anaphase I, unlike in mitosis, during which it is first quickly and then fully released [33,62,63,136]. Therefore, some substrates will remain phosphorylated between the meiotic divisions. Fission yeast retain M-CDK activity through the partial inhibition of the APC/C. The APC/C inhibitor Mes1 blocks full APC/C activity [244,245,246]. Mes1 binds and inactivates Fzr1 to prevent APC/C-Fzr1 activity by serving as a pseudo-substrate [246]. Mes1 competes as a substrate of APC/C-Slp1 to decrease the ubiquitination of Cdc13. Therefore, the levels of Cdc13 will decrease but will not be fully eliminated to allow some Cdc2-Cdc13 activity during the meiosis I-to-meiosis II transition. 

In both budding and fission yeast, the increase of cyclins in meiosis II is regulated by RNA binding proteins [51,132,143,182]. In budding yeast, the Rim4 amyloid-like translational repressor binds *CLB3* mRNA and is targeted for autophagic degradation in meiosis II, releasing *CLB3* mRNA for translation, ultimately resulting in an increase in Clb3 protein and Cdk1-Clb3 activity [145]. In fission yeast, the Spo5 RNA binding protein protects and maintains *cdc13* mRNA until meiosis II [182]. Cdc13 levels rise through de novo synthesis at the entrance into meiosis II. The current model is that Spo5 protects *cdc13* mRNA and promotes its translation in meiosis II. The increase in CDK activity allows meiosis II spindle formation and chromosome attachment. 

As cells undergo anaphase II the cyclins are targeted for degradation. In budding yeast, APC/C-Cdc20 and APC/C-Ama1 target the cyclins and other meiotic regulators for degradation, allowing meiotic exit [64]. The Cdc14 phosphatase is released from the nucleolus and removes phosphorylation from CDK substrates [62,63,253]. In fission yeast, APC/C-Slp1 and APC/C-Fzr1 target cyclins for degradation [184,185,186,187,246]. Clp1 likely reverses the phosphorylation of CDK substrates. 

Interestingly, both the meiosis-specific APC/C-Ama1 and APC/C-Fzr1 have key roles in ensuring two and only two meiotic divisions. In budding yeast, if autophagy is inhibited and Rim4 is not targeted for degradation, cells fail to exit meiosis and instead undergo additional rounds of spindle assembly, spindle elongation, and chromosome segregation [145]. *AMA1* mRNA is also a target of Rim4, and inhibition of degradation of Rim4 likely prevents an increase in Ama1 levels and APC/C-Ama1 activity [132,143]. Induced expression of *AMA1* in meiosis II suppresses the extra divisions and allows cells to exit meiosis after meiosis II [145]. Thus, APC/C-Ama1-mediated degradation of M phase proteins plays a critical role in meiotic exit. In fission yeast, deletion of *fzr1* prevents cells from exiting meiosis II and results in an additional round of chromosome segregation [186]. The results suggest that a failure to degrade key substrates at the end of meiosis II prevents meiotic exit. Additionally, the transcription factor Cuf2 increases expression of *fzr1* during meiosis II. Loss of Cuf2 results in an additional round of chromosome segregation, although the defect is not as severe as loss of Fzr1. Therefore, mechanisms in both budding and fission yeast ensure the production of specific co-activators of the APC/C to limit the number of rounds of chromosome segregation by promoting meiotic exit after meiosis II.

## 5. Future Directions 

The investigation of CDK regulation in meiosis in budding and fission yeast has greatly improved our understanding of how key meiotic events are orchestrated. Furthermore, research on CDK activity in meiosis has uncovered additional layers of regulation not found in the mitotic cell cycle. Nevertheless, further work is needed to identify a comprehensive list of meiotic Cdk1 substrates and to determine how phosphorylation regulates protein function. While we assume that for some events in meiosis, Cdk1 phosphorylates similar substrates as in the mitotic cell cycle, further investigation of meiosis-specific events is warranted. An exciting future direction would be to determine how CDK coordinates multiple events in prophase I, such as rapid chromosome movement, DSB formation, synaptonemal complex assembly, and recombination. Although some substrates have been identified, a more extensive analysis of substrates should uncover additional meiotic regulators. Similarly, further study of how CDK regulates meiosis II and meiotic exit could uncover novel regulatory mechanisms that limit meiosis to two and only two meiotic divisions. Because cell cycle regulators are highly conserved, work from budding and fission yeast has proved to be foundational for our understanding of meiotic regulation in all eukaryotes. 

## Figures and Tables

**Table 1:** Anaphase Promoting Complex/Cyclosome (APC/C) Co-activators and their Cell Cycle Targets in Meiosis.

	APC/C Co-activator	Target(s)	Active Stage(s) of Meiosis
*S. cerevisiae*	Cdc20	Clb5 Pds1	Meta I- to Ana I transition
		Clb5 Pds1 Clb1	Meta II- to Ana II transition
	Ama1	Ndd1 Clb1 Clb4 Cdc5	Prophase I
		Clb5 Pds1	Anaphase I
		Ndt80 Cdc5 Clb3	Anaphase II/Meiotic Exit
*S. pombe*	Slp1	Cdc13 Cut2 Mes1	Meta I- to Ana I transition
		Cdc13 Cut2	Meta II- to Ana II transition
	Fzr1/Mfr1	Cdc13 Cut2	Meta II- to Ana II transition/ Anaphase II/Meiotic Exit

Clb, Cyclin B; Pds1, Securin; Cdc5, Polo-like kinase; Cut2, Securin.
